# RCC1 Expression as a Prognostic Marker in Colorectal Liver Oligometastases

**DOI:** 10.3389/pore.2021.1610077

**Published:** 2021-12-02

**Authors:** Yuxiang Deng, Long Yu, Yujie Zhao, Jianhong Peng, Yanbo Xu, JiaYi Qin, Binyi Xiao, Songran Liu, Mei Li, Yujing Fang, Zhizhong Pan

**Affiliations:** ^1^ Department of Colorectal Surgery, State Key Laboratory of Oncology in South China, Collaborative Innovation Center for Cancer Medicine, Sun Yat-sen University Cancer Center, Guangzhou, China; ^2^ Department of Radiation Oncology, Peking University Shenzhen Hospital, Shenzhen, China; ^3^ Department of Pathology, State Key Laboratory of Oncology in South China, Collaborative Innovation Center for Cancer Medicine, Sun Yat-sen University Cancer Center, Guangzhou, China

**Keywords:** prognosis, expression, RCC1, colorectal liver oligometastases, pathway

## Abstract

**Introduction:** Regulator of chromatin condensation 1 (RCC1) is a major guanine-nucleotide exchange factor for Ran GTPase, and it plays key roles in various biological processes. Previous studies have found that RCC1 may play a role in the development of tumors, but little is known about the relationship between RCC1 and colorectal liver oligometastases (CLOs).

**Methods:** One hundred and twenty-nine pairs of matched human CLO samples, including both primary tumor and its liver metastasis specimens, were subjected to immunohistochemistry to determine the location and expression levels of RCC1. Associations between RCC1 and survival as well as gene expression profiling were explored.

**Results:** In this study, we first observed that RCC1 was mildly increased in CLO tumor tissues compared with normal tissues, and the localization was primarily nuclear. In addition, our study found that high RCC1 expression in liver oligometastases was an independent prognostic marker for unfavorable recurrence-free survival and overall survival (*p* = 0.036 and *p* = 0.016). Gene expression profiles generated from microarray analysis showed that RCC1 was involved in pathways including “Myc targets,” “E2F targets” and “DNA repair” pathways.

**Conclusion:** Our data indicated that RCC1 was expressed mainly in the nucleus, and strong and significant associations were found between RCC1 expression levels and the survival of CLO patients. These findings indicated that RCC1 may play a role in CLO development.

## Introduction

Colorectal cancer (CRC) is one of the most common malignant tumors in China and worldwide [1, 2]. The 5-years overall survival rate for CRC patients with early-stage disease is ~90%, but it significantly drops to ~10% for advanced-stage CRC [3, 4]. The liver is the most common metastatic site for CRC. Liver metastasis is found in 15–25% of CRC patients at the time of diagnosis [5]. The 2016 ESMO (European Society for Medical Oncology) guidelines divided metastatic CRC into two categories: oligometastatic disease and widespread systemic disease [6]. Oligometastasis is an intermediate state between localized primary tumors and widespread metastatic tumors and is a relatively early stage of biological invasion. Effective control of tumor progression, such as liver resection, can significantly prolong the survival of patients with colorectal liver oligometastases (CLOs), leading to a 5-years overall survival (OS) rate of 45.9% [7]. For those patients, the biological process of tumor development may be different. Using appropriate treatment strategies may lead to better treatment results. Therefore, exploring new biomarkers to identify high-risk patients and clarifying the underlying mechanisms are highly warranted.

Regulator of chromatin condensation 1 (RCC1) was first identified during premature chromosomal condensation in BHK cells and is known as a chromatin-bound guanine nucleotide exchange factor for the Ras-related nuclear (RAN) protein [8]. Phosphorylation of RCC1 is a key step in spindle assembly during mitosis [9].

The relationship between RCC1 and tumors has been explored. However, the role of RCC1 in tumors is controversial. RCC1 was first identified as being overexpressed in mantle-cell lymphoma by proteomic analysis [10]. Previous studies also showed statistically significantly higher RCC1 expression in ovarian tumors [11], colorectal cancer [12], carboplatin-resistant cervical tumors [13], and lung adenocarcinoma compared to normal tissues [14]. These results suggest that RCC1 may promote cancer formation. However, proteomic profiling revealed that RCC1 was decreased in HepG2 hepatoma cells induced with 6-bromine-5-hydroxy-4-methoxybenzaldehyde [15]. Another report showed that RCC1 expression was significantly lower in gastric carcinoma tissues and that the silencing of RCC1 could induce tumorigenesis and was correlated with deeper invasion in gastric cancer, indicating that RCC1 may be a tumor suppressor in gastric carcinoma [16]. There are also controversies regarding the association of RCC1 expression and survival. A tissue microarray showed that the expression of RCC1 was significantly associated with a longer overall survival of patients with low-grade B-cell lymphoma [17]. However, a survival analysis showed the opposite result: high expression of RCC1 was associated with a poor prognosis of non-small cell lung cancer [18]. All of these results indicate that the differential expression and function of RCC1 may depend on the type of tumor.

The first aim of this work was to use immunohistochemistry (IHC) to clarify the location of RCC1 in CLO tissues. RCC1 protein expression levels were evaluated in both primary tumor and its liver metastasis. Second, the role of RCC1 in predicting survival in patients with CLO was explored. Finally, we aimed to confirm the mechanisms and counteractions of RCC1 in regulating the development of CLO.

## Materials and Methods

### Patients

Retrospective study was performed on 129 consecutive CLO patients who underwent primary tumor and its liver metastasis resection from January 2004 to December 2016. The study was conducted in accordance with the Declaration of Helsinki (as revised in 2013). The inclusion criteria included the following: 1) histologically confirmed colorectal adenocarcinoma; 2) colorectal single liver metastasis; 3) R0 resection for both primary tumor and its liver metastasis; and 4) a minimum follow-up duration of 3 months. Tumor metastasis (lymph node metastasis, distant tissue and organ metastasis) and recurrence were confirmed according to radiographic results. Deaths were confirmed by consulting the patients’ immediate family. All 129 patients were followed-up until December 2020. Tissue samples were collected from patients who had signed an informed consent form and the study was approved by the Institutional Research Ethics Committee of Sun Yat-sen University Cancer Center (approval number: GZR2020-071).

### Immunohistochemical Staining

The primary tumor and its liver metastasis specimens of all included patients were formalin-fixed, paraffin-embedded. The paraffin-embedded samples were sectioned continuously into 4 μm thick slides which were then put into an oven at 60°C for dewaxing for 1 h. Then the slides were deparaffinized in 2 baths of xylene for 10 min each and rehydrated by sequential incubation with 100, 95, 80, and 70% ethanol, 5 min for each bath. Then the slides were soaked in distilled water for 3 min and were incubated with 0.3% H_2_O_2_ solution (diluted in distilled water) for 15 min to block endogenous peroxidase activity at room temperature. After that, slides were rinsed 4 times with PBS for 2 min each. Antigen retrieval was performed by boiling the tissue slides in a microwave with citrate buffer (pH 6.0). Slides were incubated with primary antibody (RCC1, 1:1500 dilution, 22335-1-AP; Proteintech, Chicago, United States) for 1 h at room temperature. Then, we used PBS to rinse the slides 4 times for 5 min each and incubated the tissues with an anti-rabbit secondary antibody at 37°C for 30 min (Zhongshan Golden Bridge Biotechnology, Beijing, China), and continued to use PBS to rinse the slides. Finally, the slides were stained with 3,3′-diaminobenzidine tetrahydrochloride (DAB, Dako, Glostrup, Denmark) till a brown color developed, and immersed in hematoxylin to stain the nucleus.

All results of IHC were evaluated using an established semi-quantitative approach by two independent pathologists in a blind manner. According to the intensity of the staining, the positive reaction of RCC1 was scored into four grades: 0 (negative), 1 (low), 2 (moderate) and 3 (high). The percentages of RCC1 positive cells were also scored into five grades: 0 (0%), 1 (5–24%), 2 (25–49%), 3 (50–74%) and 4 (75–100%). The immunoreactive score (IRS) gives a range of 0–12 as a product of multiplication between the intensity and percentage scores. The cutoff value for the IHC score for the primary tumor and liver metastasis was defined as the median value of the IHC scores. High RCC1 expression was defined as an IHC score that exceeded the cutoff value.

### Gene Expression Profiling With Microarray Analysis

The microarray experiments were conducted using an Affymetrix GeneChip Human Transcriptome Array 2.0 (HTA 2.0) (Affymetrix, Santa Clara, CA). The Affymetrix HTA 2.0 contained approximately 67,000 transcript clusters and 573,000 probe-selection regions. Thirty primary tumor tissues and 30 paired normal tissues were collected from 30 colorectal cancer patients, and RNA was isolated from each fresh tissue samples. The RNA integrity number was determined by inspecting the RNA integrity with an Agilent Bioanalyzer 2100 (Agilent Technologies, Santa Clara, CA, United States). RNA with an RNA-integrity number>7 was considered to be of suff further purified using the RNeasy Micro Kit and the RNase-Free DNase Set (both from Qiagen; GmBH, Germany). Then the RNA samples were amplified using a WT PLUS Reagent kit, followed by hybridization to HTA 2.0 microarray chips. The raw data from the HTA 2.0 chips were subjected to a quality control examination according to the Affymetrix manual. The chips that met the quality control criteria were further analyzed with a commercial software program named Partek (Partek, St. Louis) which was specifically used for microarray data analysis.

We calculated the fold change and adjusted *p*-value by the DESeq2 package for RNA-seq data, in which an adjusted *p*-value less than 0.05 was considered a differentially expressed gene (DEG). Volcano plots and heatmaps were used to display the DEGs by the R packages “ggplot2” and “pheatmap.” In addition, the “clusterProfile” package was used to perform pathway enrichment analysis based on the DEGs. The HALLMARK pathways were derived from www.gsea-msigdb.org/gsea/msigdb/index.jsp.

### Statistical Analysis

Statistical analyses were performed with SPSS 20.0 (Chicago, IL, United States), R (version 4.0.2, R foundation for statistical, Vienna, Austria), and GraphPad Prism 7 software (La Jolla, CA, United States). Categorical variables are presented as percentages, and categorical variables were compared using the chi-square (χ^2^) test or nonparametric Spearman’s correlation test. The Kaplan–Meier method was used to construct the survival and recurrence curves. Cox proportional hazards model analysis was used to analyze the correlation between variables and CLO patient prognosis. Statistical tests were two-tailed, and *p*-values <0.05 were considered significant.

## Results

### Patient Characteristics

Baseline clinical demographics and laboratory values are presented in Table 1. With a median follow-up time of 63 months (range, 4–177 months) after liver resection, 49 (38.0%) patients experienced cancer-related mortality, and 61 (47.3%) patients experienced disease recurrence. The right-sided CRC tumors arise from cecum, ascending colon, and proximal two thirds of the transverse colon and the left-sided CRC tumors arise from the descending, sigmoid colon, rectum, and distal one third of the transverse colon.

**TABLE 1:** Clinical characteristics of 129 patients with colorectal liver oligometastases.

Parameters	Total patients (n, %)
Median age (years)	58 (25–77)
Age, years	
≤60	75 (58.1)
>60	54 (41.9)
Gender	
Female	53 (41.1)
Male	76 (58.9)
Primary tumor location	
Right-sided	32 (24.8)
Left-sided	97 (75.2)
Primary tumor differentiation	
Well/Moderate	95 (73.6)
Poor	34 (26.4)
T stage	
1	1 (0.8)
2	11 (8.5)
3	71 (55.0)
4	33 (25.6)
Not available	13 (10.1)
N stage	
0	40 (31.0)
1	51 (39.5)
2	23 (17.8)
Not available	15 (11.6)
Liver metastases tumor size (cm)	
Median (range)	2.2 (0.5–8.7)
≤2.2	65 (50.4)
>2.2	64 (49.6)
Hepatic resection timing	
Synchronous	72 (55.8)
Metachronous	57 (44.2)
Preoperative CEA (ng/ml)	
≤5	49 (38.0)
>5	80 (62.0)
Preoperative CA19-9 (U/ml)	
≤35	91 (70.5)
>35	38 (29.5)
Preoperative chemotherapy	
FOLFIRI	9 (7.0)
FOLFOX	12 (9.3)
XELOX	10 (7.8)
XELODA	3 (2.3)
No	95 (73.6)
Adjuvant chemotherapy	
FOLFIRI	16 (12.4)
FOLFOX	21 (16.3)
XELOX	42 (32.6)
XELODA	7 (5.4)
No	43 (33.3)
RCC1 expression of liver oligometastases	
Low	55 (42.6)
High	74 (57.4)

TNM stage, tumor-node-metastasis classification; CEA, carcinoembryonic antigen; CA19-9, carbohydrate antigen 19-9.

### RCC1 Staining Scores in Tumor and Normal Tissues

**FIGURE 1:**
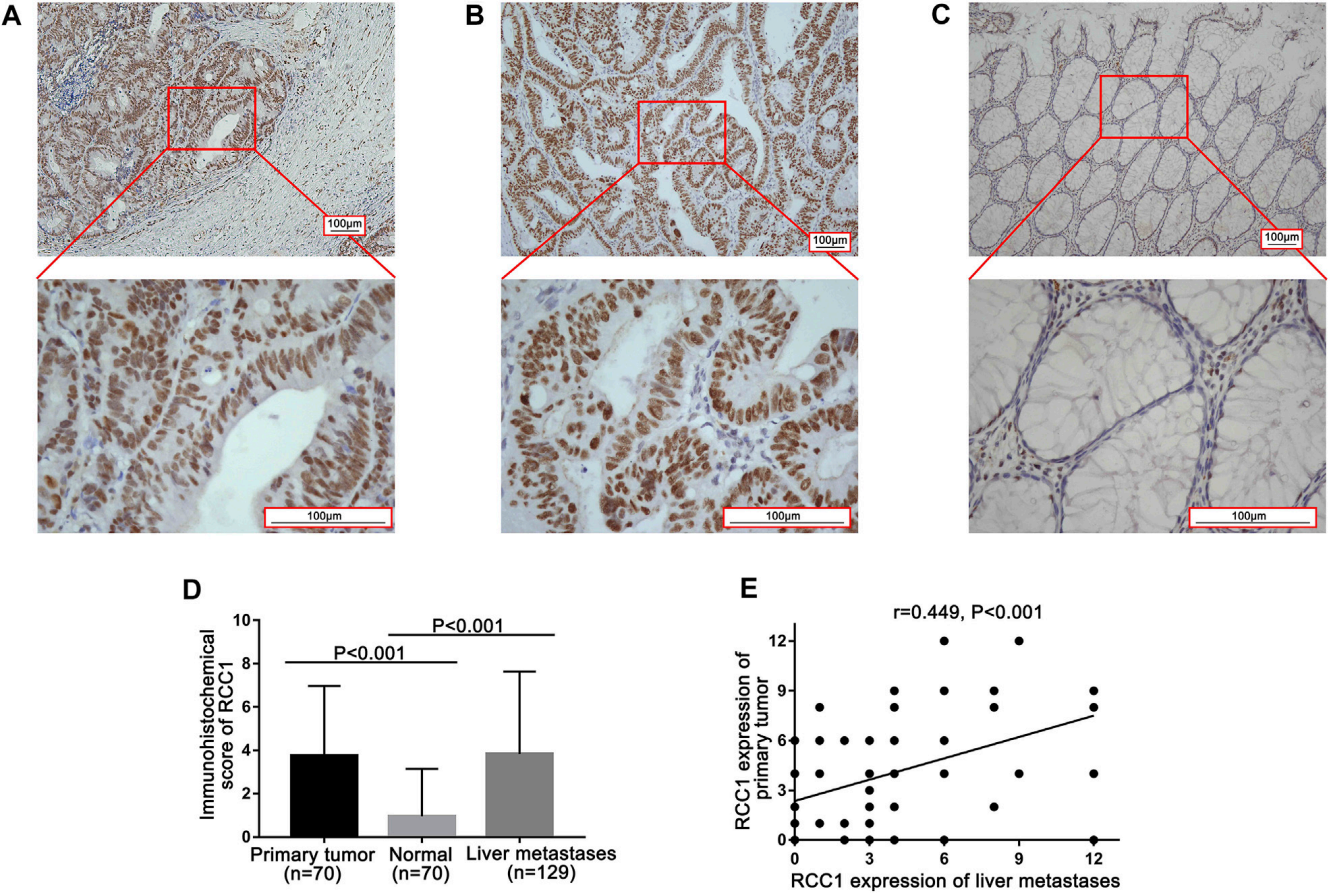
RCC1 expression in the primary tumor and paired normal tissue as well as its liver metastasis by immunohistochemistry (IHC). **(A)** RCC1 expression in primary tumor. **(B)** RCC1 expression in normal tissue. **(C)** RCC1 expression in liver metastases. The original magnifications were ×100 and ×400 with a 100 μm scale. **(D)** Comparison of RCC1 expression among primary tumors, normal tissue, and liver metastases. **(E)** Correlation of RCC1 expression levels between primary tumors and liver metastases.

In 129 patients, RCC1 expression was detected in 70 primary tumors and matched normal tissues as well as in 129 liver metastases. Fifty-nine primary tumor specimens were not accessible because the patients had operations in other hospitals. RCC1 was expressed in the nucleus of glandular cells in the primary tumor, normal tissues and liver oligometastases (Figures 1A–C). Positive RCC1 expression was observed in 81.4% (57/70) of primary tumor tissues, 18.6% (13/70) of normal tissues, and 71.3% (92/129) of liver oligometastatic tissues. The levels of RCC1 in the primary tumors and liver metastases were significantly higher than those in normal tissues (Figure 1D), and a significant positive correlation of RCC1 expression was noted between primary tumors and liver metastases (r = 0.449, *p* < 0.001; Figure 1E).

### Relationship of RCC1 Staining Scores and Clinicopathological Features

To determine the prognostic value of RCC1, CLO cohorts were divided into high expression (IHC score ≥ 3) and low expression (IHC score < 3) groups with a median cut-off and Kaplan-Meier analyses were performed between the groups. The correlation between RCC1 expression in liver metastasis and clinicopathological features had been presented in Table 2 and no significant association was found. Similarly, no significant correlation was found between the expression level of RCC1 in primary tumors and clinicopathological characteristics (Supplementary Table S1).

**TABLE 2:** Association of RCC1 expression with liver oligometastases and the clinicopathological parameters of all patients.

Parameters	Low RCC1 expression (*n* = 55, %)	High RCC1 expression (*n* = 74, %)	*p* Value
Age (years)			
≤60	35 (63.6)	40 (54.1)	0.275
>60	20 (36.4)	34 (45.9)	
Gender			
Female	22 (40.0)	31 (41.9)	0.829
Male	33 (60.0)	43 (58.1)	
Primary tumor location			
Right-sided	12 (21.8)	20 (27.0)	0.498
Left-sided	43 (78.2)	54 (73.0)	
Primary tumor differentiation			
Well to moderate	45 (81.8)	50 (67.6)	0.069
Poor	10 (18.2)	24 (32.4)	
T stage			
1–3	38 (69.1)	45 (60.8)	0.355
4	12 (21.8)	21 (28.4)	
Not available	5 (9.1)	8 (10.8)	
N stage			
0	18 (32.7)	22 (29.7)	0.749
1–2	31 (56.4)	43 (58.1)	
Not available	6 (10.9)	9 (12.2)	
Liver metastases tumor size (cm)			
≤2.2	30 (54.5)	35 (47.3)	0.415
>2.2	25 (45.5)	39 (52.7)	
Hepatic resection timing			
Synchronous	30 (54.5)	42 (56.8)	0.802
Metachronous	25 (45.5)	32 (43.2)	
Preoperative CEA (ng/ml)			
≤5	16 (29.1)	33 (44.6)	0.073
>5	39 (70.9)	41 (55.4)	
Preoperative CA19-9 (U/ml)			
≤35	39 (70.9)	52 (70.3)	0.937
>35	16 (29.1)	22 (29.7)	
Preoperative chemotherapy			
Yes	14 (25.5)	20 (27.0)	0.841
No	41 (74.5)	54 (73.0)	
Adjuvant chemotherapy			
Yes	37 (67.3)	49 (66.2)	0.900
No	18 (32.7)	25 (33.8)	

TNM stage, tumor-node-metastasis classification; CEA, carcinoembryonic antigen; CA19-9, carbohydrate antigen 19-9.

### Association of RCC1 Staining Scores and Clinical Outcomes

**FIGURE 2:**
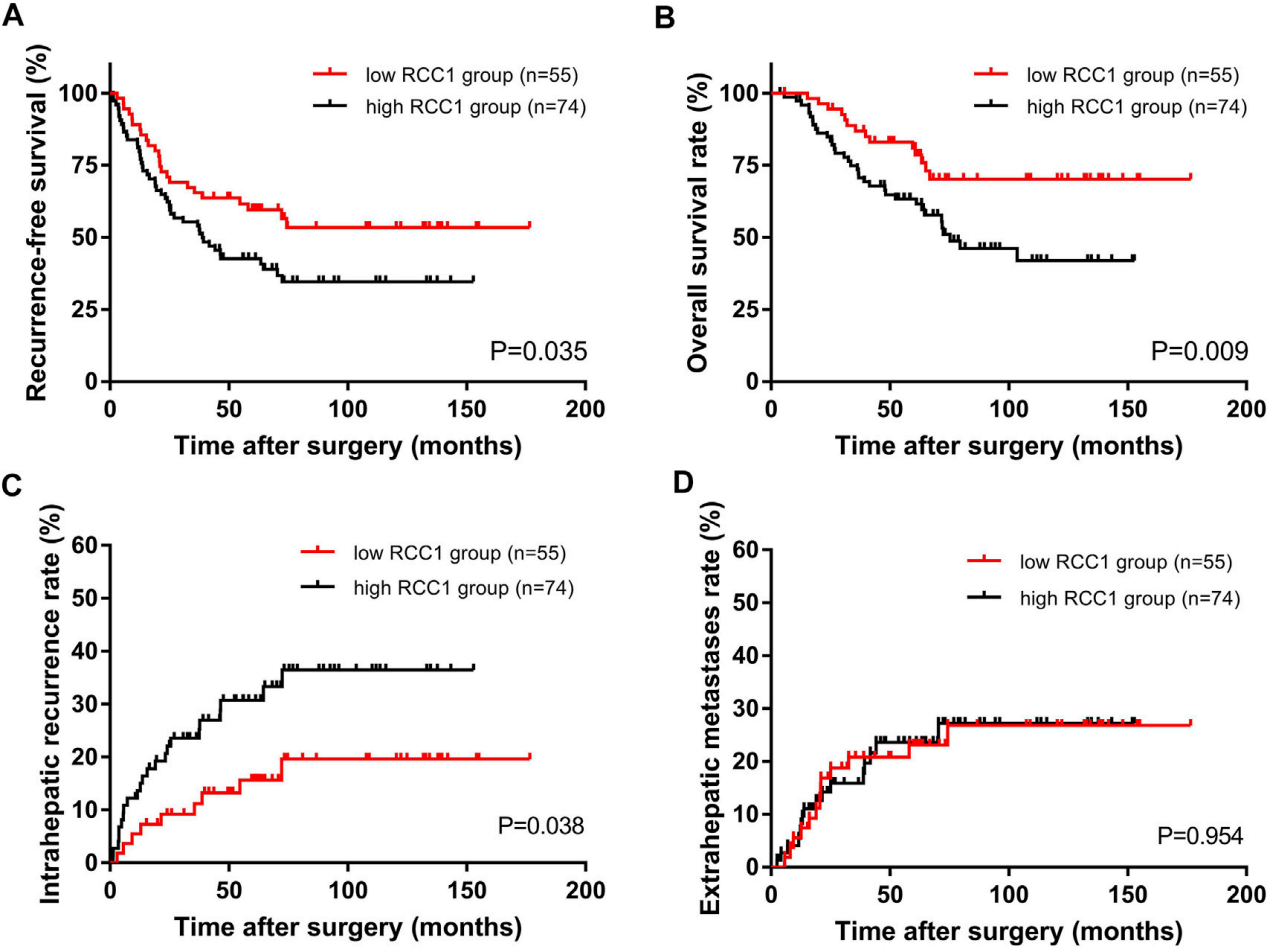
Kaplan–Meier long-term survival curves grouped by high and low RCC1 expression in CLO patients. **(A)** RFS rate and **(B)** OS rate comparison analysis of patients with high and low RCC1 expression in liver metastases. **(C)** Cumulative incidence of intrahepatic recurrence and **(D)** extrahepatic metastasis in the high and low RCC1 expression groups.

The total recurrence-free survival (RFS) rates for all patients at 1, 3, and 5 years were 84.5, 59.6, and 49.8%, and the OS rates were 98.4, 80.0, and 70.8%, respectively. Kaplan-Meier survival analysis showed that the median RFS times of the high RCC1 group and the low RCC1 group were 38.3 and 60.5 months, respectively; the median OS times were 58.4 and 63.7 months, respectively. The 1-, 3-, and 5-years RFS rates for the high and low RCC1 groups were 81.1 and 89.1%, 55.3 and 65.5%, 42.6 and 59.5%, respectively. The OS rates at 1, 3, and 5 years were 97.3 and 100%, 74.9 and 86.9%, and 63.3 and 80.9%, respectively. The differences in RFS and OS were statistically significant (*p* = 0.035, Figure 2A; *p* = 0.009, Figure 2B). Regarding the cumulative incidence of postoperative recurrence, the 1-, 3-, and 5-years cumulative intrahepatic recurrence rates were 13.6 and 5.5%, 23.6 and 11.2%, 30.7 and 15.6% for the high- and low-RCC1 expression groups, respectively (*p* = 0.038, Figure 2C). The 1-, 3-, and 5-years cumulative extrahepatic metastases rates were 5.5 and 5.6%, 15.9 and 20.8%, 23.6 and 23.1% for the high- and low-RCC1 expression groups, respectively (*p* = 0.954, Figure 2D).

Univariate Cox regression analysis revealed that N stage (*p* = 0.006; *p* = 0.005), hepatic resection timing (*p* = 0.042; *p* = 0.008) and the RCC1 level in liver oligometastases (*p* = 0.037; *p* = 0.011) were associated with RFS and OS. In addition, perioperative chemotherapy (*p* = 0.040) was associated with RFS (Table 3).

**TABLE 3:** Univariate and multivariate analyses of the factors influencing OS and RFS with a Cox proportional hazard model.

Parameters	RFS				OS			
	Univariate analysis		Multivariate analysis		Univariate analysis		Multivariate analysis	
	HR (95% CI)	*p* Value	HR (95% CI)	*p* Value	HR (95% CI)	*p* Value	HR (95% CI)	*p* Value
Age (year)								
>60 vs. ≤60	0.702 (0.430–1.145)	0.157			0.915 (0.515–1.626)	0.762		
Gender								
Male vs. Female	0.914 (0.570–1.468)	0.711			0.972 (0.552–1.712)	0.921		
Primary tumor location								
Left-sided vs Right-sided	1.216 (0.696–2.126)	0.492			1.524 (0.738–3.144)	0.255		
Primary tumor differentiation								
Well/moderate vs Poor	1.159 (0.684–1.964)	0.582			1.190 (0.640–2.213)	0.582		
T category								
4 vs. 1–3	1.472 (0.881–2.459)	0.140			1.287 (0.684–2.419)	0.434		
N category								
1–2 vs. 0	2.199 (1.249–3.874)	0.006	2.246 (1.274–3.959)	0.005	3.024 (1.403–6.516)	0.005	3.099 (1.437–6.680)	0.004
Liver metastases tumor size (cm)								
>2.2 vs. ≤2.2	1.028 (0.643–1.645)	0.907			0.989 (0.563–1.739)	0.970		
Hepatic resection timing								
Synchronous vs. Metachronous	1.647 (1.019–2.663)	0.042	0.764 (0.461–1.267)	0.297	2.240 (1.237–4.057)	0.008	0.0531 (0.277–1.016)	0.056
Preoperative CEA (ng/ml)								
>5 vs. ≤5	1.054 (0.649–1.713)	0.832			1.183 (0.650–2.154)	0.582		
Preoperative CA19-9 (U/ml)								
>35 vs. ≤ 35	0.912 (0.543–1.532)	0.727			1.243 (0.690–2.239)	0.469		
Perioperative chemotherapy								
Yes vs. No	1.879 (1.028–3.433)	0.040	1.613 (0.842–3.092)	0.150	1.670 (0.810–3.443)	0.165		
RCC1 expression of liver metastasis								
High vs. Low	1.692 (1.032–2.774)	0.037	1.726 (1.037–2.873)	0.036	2.227 (1.197–4.144)	0.011	2.223 (1.162–4.253)	0.016

OS, overall survival; RFS, recurrence-free survival; HR, hazard ratio; CI, confidence interval; CEA, carcinoembryonic antigen before liver tumor resection; CA19-9, carbohydrate antigen 19-9 before liver tumor resection.

Multivariate analysis showed that N+ (HR, 2.246; 95% CI, 1.274–3.959; *p* = 0.005) (HR, 3.099; 95% CI, 1.437–6.680; *p* = 0.004) and high RCC1 expression in liver oligometastases (HR, 1.726; 95% CI, 1.037–2.873; *p* = 0.036) (HR, 2.223; 95% CI, 1.162–4.253; *p* = 0.016) were independent prognostic markers for unfavorable RFS and OS.

### Associations Between the Gene Expression Profiles and RCC1 Expression

**FIGURE 3:**
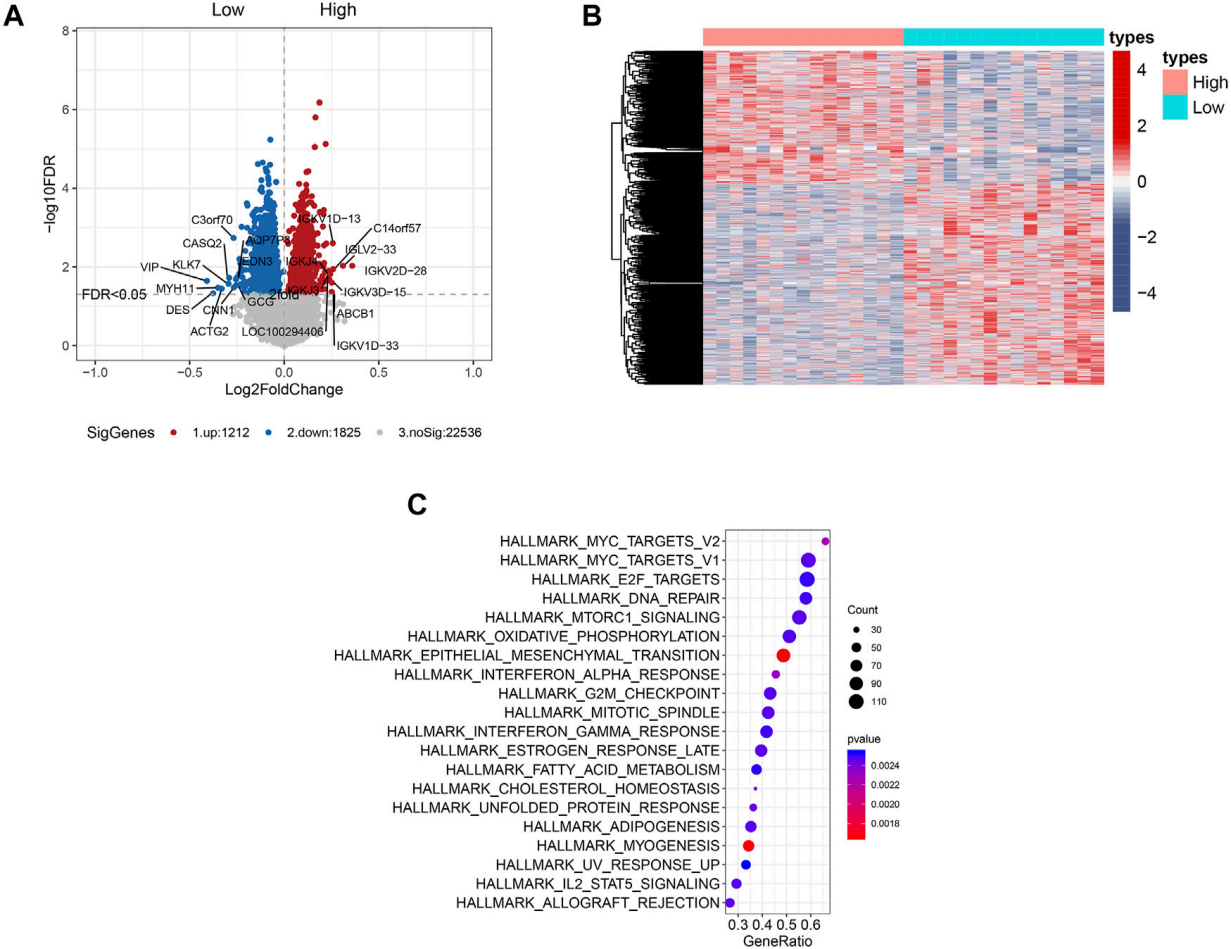
Genome-wide gene expression profile and the signaling pathways associated with RCC1 expression. **(A)** Volcano plot of some of the significantly up- and downregulated genes with differential RCC1 expression. Significantly regulated genes associated with high RCC1 expression and low RCC1 expression are marked by red and blue circles, respectively. **(B)** Expression heatmap of RCC1 expression. **(C)** HALLMARK pathway analysis of the cell signaling pathways related to RCC1 expression.

The clinical characteristics of the patients who were selected for gene expression profiling with microarray analysis are presented in Supplementary Table S2. Gene expression profiling identified multiple genes that were significantly associated with RCC1 expression (*p* < 0.05, Figure 3A), either in positive or negative ways. The top 5 upregulated genes include VIP, DES, MYH11, ACTG2 and KLK7. The top 5 downregulated genes include IGKV2D-28, REG3A, IGKV2D-30, IGLV2-33 and L1TD1. These genes are presented in the aberrant expression heat map (Figure 3B). HALLMARK pathway analysis indicated that the genes that were significantly associated with RCC1 expression were mainly involved in “Myc targets,” “E2F targets” and “DNA repair” pathways (Figure 3C). The relevant gene expression levels of the above pathways were shown in Supplementary Table S3.

## Discussion

This study provides the first evidence for RCC1 as a prognostic marker in CLO patients. In the present study, we identified the location of RCC1 through IHC and explored its association with novel pathways by microarray analysis. We showed that high RCC1 expression was more common in both primary tumors and liver metastases than in normal tissues. Moreover, our results indicated that high RCC1 expression was significantly correlated with a worse prognosis in CLO patients. These findings have important implications for CLO patients during clinical work.

CLO is a heterogeneous disease that displays various biological and clinical characteristics. As shown by our results, the crucial role of cellular RCC1 concentrations is a salient feature of CLO tissues. Interestingly, endogenous RCC1 expression in primary carcinoma cells and in liver metastases were linearly positively correlated. The underlying mechanism remains to be determined. Recently, many molecular markers have been explored to predict the outcomes of CLO patients, but their roles in determining the risk level of an individual patient are quite limited. Here, we provided extensive evidence that indicates a critical association of RCC1 with survival. Based on our data, RCC1 may play a tumor-promoting role in CLO. Therefore, it is worthy of further research.

RCC1 is a well-known protein that has a key role in the activation of proteins required for kinetochore assembly, spindle formation, or nuclear envelope formation, among other mitotic events. As a Ran-related protein, RCC1 contributes to the transformation of RanGDP into RanGTP [19]. Increased RCC1 expression could increase cellular RanGTP levels and enhance the function of importin β and exportin 1, which accelerate cell cycle progression and modulate cellular responses to DNA damage [20]. The RCC1-Ran complex acts as a component of a signal transmission pathway that detects unreplicated DNA and prevents it from entering mitosis [21]. RCC1 was initially identified as a regulator of the onset of chromosome condensation in the G1/S transition [22].

The mutation and expression level of RCC1 may be closely related to the development of tumors. In our study, RCC1 overexpression was correlated with a worse clinical outcome, but the underlying mechanism was unclear. Riahi et al. identified a novel mutation in RCC1 as a breast cancer susceptibility allele through exome sequencing that has exclusively been found in Tunisian breast cancer patients [23]. In fact, RCC1 blockade is being investigated as a potential therapeutic strategy against aggressive breast tumors [24]. Haggag et al. showed that liposome-mediated codelivery with Ran-RCC1 inhibitory peptide could improve the antitumor effect of doxorubicin in tumor-bearing mice [24]. A previous study found that downregulation of RCC1 could sensitize immunotherapy by upregulating PD-L1 via the p27kip1/CDK4 pathway in non-small cell lung cancer, and the expression of RCC1 was inversely related to the amount of immune cell infiltration [18]. It was also reported that RCC1 promotes doxorubicin resistance in colorectal carcinoma cells [20]. This study found that compared to slow-growing cells, rapidly proliferating tissue culture cells expressed approximately 4-fold higher levels of RCC1. In addition, RCC1 overexpression strongly increased cell survival following doxorubicin-induced DNA damage. RCC1 overexpression was sufficient to accelerate the cell cycle and DNA damage repair after doxorubicin treatment. Qiao et al. showed that RCC1 was upregulated by c-Jun in both cervical cancer tissues and HPV-16 E7-expressing cells. RCC1 was involved in E7-mediated abrogation of the G1 checkpoint through regulation of E2F1 degradation, and Cdk1, an E2F1 target, can rescue G1/S progression rates [25]. Deschamps T et al. identified RCC1 as a novel mediator of Epstein-Barr virus nuclear antigen 1 interaction with metaphase chromosomes. They confirmed that the interaction between EBNA1 and RCC1 was direct [26]. These findings may be related to our results that RCC1 upregulation could indicate worse survival of CLO patients.

However, many of the underlying cellular and molecular mechanisms remain to be explored. To clarify this, we performed gene expression profiling with microarray analysis. Our results indicated that RCC1 was significantly associated with the “Myc targets,” “E2F targets” and “DNA repair” pathways. Based on our results, RCC1 may be related to E2F conditions. In colorectal cancer, RCC1 may promote cell cycle progression by changing the E2F status, thereby affecting the progression of tumors. In addition, upstream RCC1 may be regulated by Myc, and altered RCC1 influences DNA damage repair, triggering tumor progression.

## Conclusion

In general, we demonstrated that RCC1, which is expressed not only in primary CRC tumors but also in liver metastases, is an important biomarker in predicting the survival of CLO patients. Additionally, our data suggest that RCC1 may be related to the “Myc targets,” “E2F targets” and “DNA repair” pathways. Taken together, this study is the first to identify a relationship between RCC1 and the prognosis of CLO patients and to reveal a possible function of RCC1 in DNA repair and E2F target pathways. These results suggested that the expression of RCC1 might play a role in tumorigenesis and in predicting survival, and further research is needed to explore the underlying mechanisms.

## Data Availability

The datasets presented in this study can be found in online repositories. The names of the repository/repositories and accession number(s) can be found below: The authenticity of this article has been validated by uploading the key raw data onto the Research Data Deposit public platform (http://www.researchdata.org.cn), with the approval number as RDDA2021001957.
