# Supplementary material for: RCC1 Expression as a Prognostic Marker in Colorectal Liver Oligometastases
Source: Pathol Oncol Res. 2021 Dec 2;27:1610077. doi: 10.3389/pore.2021.1610077 (PMC8674189; doi:10.3389/pore.2021.1610077)
Supplement: Supplementary file 1 [file Table1.docx]

**Supplemental Table 1. Association of RCC1 expression in primary tumor and the clinicopathological parameters of all patients**

| **Parameters** | **Low RCC1 expression (n=33, %)** | **High RCC1 expression (n=37, %)** | **P value** |
| --- | --- | --- | --- |
| Age (years) |  |  |  |
| ≤ 60 | 19 (57.6) | 19 (51.4) | 0.602 |
| > 60 | 14 (42.4) | 18 (48.6) |  |
| Gender |  |  |  |
| Female | 15 (45.5) | 17 (45.9) | 0.967 |
| Male | 18 (54.5) | 20 (54.1) |  |
| Primary tumor location |  |  |  |
| Right-sided | 9 (27.3) | 9 (24.3) | 0.778 |
| Left-sided | 24 (72.7) | 28 (75.7) |  |
| Primary tumor differentiation |  |  |  |
| Well to moderate | 25 (75.8) | 23 (62.2) | 0.221 |
| Poor | 8 (24.2) | 14 (37.8) |  |
| T stage |  |  |  |
| 1-3 | 24 (72.7) | 26 (70.3) | 0.820 |
| 4 | 9 (27.3) | 11 (29.7) |  |
| N stage |  |  |  |
| 0 | 7 (21.2) | 11 (29.7) | 0.459 |
| 1-2 | 25 (75.8) | 26 (70.3) |  |
| Not available | 1 (3.0) | 0 (0) |  |
| Liver metastases tumor size (cm) |  |  |  |
| ≤ 2.2 | 19 (57.6) | 18 (48.6) | 0.455 |
| > 2.2 | 14 (42.4) | 19 (51.4) |  |
| Hepatic resection timing |  |  |  |
| Synchronous | 27 (81.8) | 29 (78.4) | 0.719 |
| Metachronous | 6 (18.2) | 8 (21.6) |  |
| Preoperative CEA (ng/ml) |  |  |  |
| ≤ 5 | 13 (39.4) | 12 (32.4) | 0.544 |
| > 5 | 20 (60.6) | 25 (67.6) |  |
| Preoperative CA19-9 (U/ml） |  |  |  |
| ≤ 35 | 22 (66.7) | 26 (70.3) | 0.746 |
| > 35 | 11 (33.3) | 11 (29.7) |  |
| Preoperative chemotherapy |  |  |  |
| Yes | 9 (27.3) | 8 (21.6) | 0.582 |
| No | 24 (72.7) | 29 (78.4) |  |
| Adjuvant chemotherapy |  |  |  |
| Yes | 21 (63.6) | 28 (75.7) | 0.273 |
| No | 12 (36.4) | 9 (24.3) |  |

Abbreviations: TNM stage, tumor-node-metastasis classification; CEA, carcinoembryonic antigen; CA19-9: carbohydrate antigen 19-9.
