# Supplementary material for: RCC1 Expression as a Prognostic Marker in Colorectal Liver Oligometastases
Source: Pathol Oncol Res. 2021 Dec 2;27:1610077. doi: 10.3389/pore.2021.1610077 (PMC8674189; doi:10.3389/pore.2021.1610077)
Supplement: Supplementary file 2 [file Table2.docx]

**Supplemental Table 2. Clinical characteristics of 30 patients whose fresh colorectal cancer tissues were analyzed with a microarray**

| **Characteristics** | **N = 30 (%)** |
| --- | --- |
| Age, year (median, range) | 55 (30-70) |
| Gender |  |
| Male | 16 (53.3) |
| Female | 14 (46.7) |
| Location |  |
| Colon | 28 (93.3) |
| Rectum | 2 (6.7) |
| T stage |  |
| 1 | 3 (10.0) |
| 2 | 4 (13.3) |
| 3 | 15 (50.0) |
| 4 | 8 (26.7) |
| N stage |  |
| 0 | 15 (50.0) |
| 1 | 10 (33.3) |
| 2 | 5 (16.7) |
| TNM stage |  |
| Ⅰ | 6 (20.0) |
| Ⅱ | 6 (20.0) |
| Ⅲ | 6 (20.0) |
| Ⅳ | 12 (40.0) |

Abbreviations: TNM stage, tumor-node-metastasis classification
