# Supplementary material for: RCC1 Expression as a Prognostic Marker in Colorectal Liver Oligometastases
Source: Pathol Oncol Res. 2021 Dec 2;27:1610077. doi: 10.3389/pore.2021.1610077 (PMC8674189; doi:10.3389/pore.2021.1610077)
Supplement: Supplementary file 3 [file Table3.docx]

**Supplemental table 3. "Myc targets", "E2F targets" and "DNA repair" associated genes that were related to the expression of RCC1 in the microarray.**

| Gene symbol | Log_2_ Fold change |
| --- | --- |
| MYC | 0.128 |
| CDK4 | 0.113 |
| CCNB2 | 0.102 |
| CDK1 | 0.091 |
| CDKN3 | 0.086 |
| ERCC1 | 0.080 |
| CDK2 | 0.077 |
| CDC25A | 0.072 |
| CHEK2 | 0.068 |
| CDKN1B | 0.064 |
| CHEK1 | 0.063 |
| CDK6 | 0.060 |
| JUN | 0.049 |
| CDKN1A | 0.045 |
| TP53 | 0.043 |
| BRCA1 | 0.039 |
| ATM | 0.022 |
| ATR | -0.003 |
| CCND1 | -0.004 |
| ATRX | -0.009 |
| CD274 | -0.012 |
| BRCA2 | -0.067 |
